# Letter from Jesse W. Cook, M.D., D.D.S., to the Baltimore Editor

**Published:** 1844-09

**Authors:** Jesse W. Cook

**Affiliations:** Cincinnati, Ohio


### ARTICLE IV.

### *Letter from Jesse W. Cook, M. D., D. D. S. to the Baltimore Editor.*

*Dear Friend:*

In compliance with my promise, I herein transmit to you a statement of an operation performed by Professor Mussey and myself, on young Mr. Coudon, of Warren Co., Ohio, in April, 1841.

Age 18.—The nature of the case was congenital complicated hare-lip, cleft palate, deficient palatine arch and nasal septum, posteriorly, running down, anteriorly, to a small nipple-like cartilaginous projection. That portion of the superior maxillary bones between the cuspidatus, was entirely wanting. After the above description, you can well imagine the hideousness of his appearance, and want of articulation. I could not understand a single word he expressed, without the assistance of his brother, as interpreter, who understood him pretty well.

Without giving a minute description of the operation on the soft parts, by Professor Mussey, suffice it to say, that the superior labia was beautifully united up to the nose, leaving but a small cicatrix. The soft palate lay like inclined vertical folds of soft tissue, on the posterior lateral walls of the fauces, from whence they were lifted, brought together, and secured by suture, and that also united from point to point, making a perfect palate. We now have a lip and a palate, with the countenance much improved, and a smile on the cheek, with glistening hope of future happiness beaming from his eyes, the happy result of scientific surgery. Though much improved, not yet is the organism of the mouth complete.

First part of dental operation: I took a perfect impression in wax, of the upper half of the mouth, nasal cavities and septum above, taking care to observe a proper degree of rotundity and elevation forward, as a base for the jaw and processes of the four superior incisor teeth.

2d. I then made a cast of calcined plaster.

3d. Two male and female casts of zinc and lead.

4th. I rolled out a plate of fine gold, large enough to overlap

ARMY MEDICAL LIBRARY

the entire cast an inch all round, and much thicker than we commonly use for plate work.

5th. One of the male casts was taken in the cavity, within the bounds of the teeth and soft palate, up to, and in close contact with, that portion of the nose as described; with which I drove up into one of the leaden female casts the plate, perfectly adapting it to the singular structure therein; making a groove the whole length for the septum narium to rest in air-tight.

6th. The plate, as altered by the first process, after being well annealed, (which was frequently observed,) was placed in the other leaden female cast, and drove up with its fellow male, bringing every part of the lateral overlapping edges of the plate into close contact with the teeth. The plate on its upper surface, as now described, forms the deficient partition and floor of the nasal cavities, its under surface forming an irregular concavity or roof of the mouth.

7th. For the purpose of forming a palatine arch, to represent nature's own perfect work, and guard against the accumulation of foreign matter in the cavity described, I filled it with wax, observing the natural concavity and shape of the mouth, within the bounds of the teeth on the sides; forward, where I contemplated the alveolar processes and incisor teeth to be connected, I cut the wax up from its palatine surface, observing the natural formation of the jaw, until I came in contact with the superior plate.

8th. A wall of wax was kept built up two and a half inches around it, in which I took a plaster cast, followed with a zinc male and leaden female cast, into which another gold plate was driven up, forming the representative of the natural roof, and overlapping the anterior space between the upper and lower plates. After filing and bevelling up the edges, I found it a perfect fit.

9th. The next process was in soldering them together, adjusting broad clasps around the molars on each side, setting the incisors on the front, and finishing.

In putting into the mouth the piece of mechanism now constructed, we usher into existence almost a new creature, changed, at least, much from the revolting appearance presented at our first introduction. Where vacuity was perceptible, and the want of substance marked its exterior margin, we now behold with pleasure, the gems of pearly lustre shining forth in all their beauty at every smile, from under his lips of ruby hue. No longer is the ear pained at the uncouth, discordant sounds rolling from the great cleft of mal-formation; his articulation and pronunciation much improved, like others of his age now, with the same opportunities, speaks to be understood.

As an evidence of the change produced in his mind, on looking into the glass after its application, he observed, "I will now have a chance to get a wife." What a revolution of feeling! what pleasing sensations! what a powerful transition from the hopeless state of despair, to the probable chance of success, to possess and enjoy one of God's greatest blessings to man here below—a wife.

It is with pleasure that I am enabled to inform the profession that, from the period of its introduction up to the present time, August 16th, 1844, there has not been the slightest irritation or inconvenience arising from its use; he enjoys good health, and a free flow of spirits.

JESSE W. COOK.

*Cincinnati, Ohio, Aug. 16th,* 1844.

